# Diabetes during the COVID-19 pandemic: are people getting access to the right level of care?

**DOI:** 10.1186/s12913-023-09168-2

**Published:** 2023-02-16

**Authors:** Gideon Meyerowitz-Katz, Shahana Ferdousi, Glen Maberly, Thomas Astell-Burt

**Affiliations:** 1 Western Sydney Local Health District, Blacktown Hospital, 18 Blacktown Road, Blacktown, NSW 2148 Australia; 2 Population Wellbeing and Environment Research Lab, School of Health and Society, Faculty of Arts, Social Sciences and Humanities, University of Wollongong, Wollongong, NSW Australia; 3 Wentwest, Western Sydney Primary Health Network, Campbelltown, NSW Australia; 4 Boden Initiative, Charles Perkins Centre, University of Sydney, Camperdown, NSW Australia; 5 Menzies Centre for Health Policy and Economics, University of Sydney, Camperdown, NSW Australia

**Keywords:** Diabetes, Epidemiology, COVID-19, Chronic Disease, Population Health

## Abstract

**Introduction:**

Avoidance of health services, in particular hospital and community services, is problematic for people with diabetes. Evidence has demonstrated that such missed attendances are associated with worse health, faster declines in functioning, and higher rates of mortality long-term.

This paper investigated the impact of the pandemic on healthcare access across community and hospital care, including Virtual Care (VC) using several large datasets of General Practice (GP) and hospital services in western Sydney.

**Methods:**

A retrospective cohort study using a time-series database of 173,805 HbA_1c_ tests done at Blacktown and Mt Druitt hospitals and 1.8 million recorded consultations at GP clinics in the region was undertaken.

**Results:**

The average rate of diabetes in Emergency Department fell from 17.8% pre-pandemic to 11% after January 2020 (*p* < 0.001). This rate varied substantially over time, and correlated well with large outbreaks of COVID-19 in the state. Conversely, attendances of people with diabetes to GP clinics, especially using VC services, increased substantially over the pandemic period.

**Discussion/Conclusion:**

During the pandemic there was a substantial avoidance of hospital care by patients with diabetes. However, this may have been replaced by VC offered in the community for those with less severe diseases.

## Introduction

The emergence of COVID-19 as a global pandemic has dramatically changed the way healthcare has been delivered. In preparation for large upsurges in patient numbers requiring care, people were originally discouraged from attending healthcare services at the beginning of 2020. Moreover, people with diabetes were quickly identified as having a very significantly increased risk from COVID-19, with higher risks of hospitalization and death if they catch the disease [1]. The ongoing fear of COVID-19, particularly for high-risk people, has also been demonstrated to have impacted their movement [2, 3] and likelihood of seeking care for medical issues. Moreover, there is evidence that lockdowns have been associated with reduced access of care in many situations, despite the lockdown itself not necessarily impeding the ability to attend a medical institution [4].

Western Sydney is a moderately large area in the west of Sydney, Australia, encompassing roughly 1 million inhabitants [5]. It is one of the most culturally diverse places in Australia, with nearly 60% of inhabitants having been born overseas [5], and it also has the numerically largest population of Aboriginal people of any local health area in Australia. The region is also home to very disadvantaged neighbourhoods, including the most disadvantaged postcode in the city of Sydney. Given these characteristics, Western Sydney is a diabetes ‘hotspot’ with rates of diabetes double that of the less disadvantaged areas in the city [6, 7].

To improve care in this locality, routine testing to detect diabetes in patients attending the Emergency Department (ED) has been established in some of the local hospitals [8]. Using this protocol, HbA_1c_ testing is undertaken in all patients over the age of 18 who had blood sampled in ED. This protocol confirmed the findings of high rates of diabetes undertaken in a previous study [9]. A recent study looking at a similar protocol in nearby community general practice clinics identified a similar burden of diabetes in this setting, with the rate of HbA_1c_ consistent with diabetes estimated at 17% in both of these studies [8]. HbA_1c_ levels consistent with pre-diabetes based on American Diabetes Association criteria [10] was found to be 30% using this testing protocol. Since the start of the hospital testing methodology, over 170,000 tests have been performed on more than 100,000 patients, providing a large dataset for analyses in this study.

Another extremely valuable source of information in this local area is the accumulation of general practice data in aggregate form by the primary health network, Wentwest. This represents the de-identified data of all patients attending 188 general practices (GP) across the region. While not as granular as the testing information from EDs, this dataset is extremely large, with close to 2 million patients – including historical data—included overall.

Another useful facet of the healthcare relationship to consider is the potential switch from face-to-face (FTF) services during the pandemic to virtual care (VC) modalities. During times where social distancing was of key importance and health services often could not offer FTF services, VC became the primary modality through which patients with diabetes accessed ongoing services for their chronic condition [11]. Where previously VC was one option of many, it became the method of access for most outpatient and ongoing healthcare services during both lockdowns and periods of high transmission.

This study presents an analysis of the rate of HbA_1c_ tests consistent with diabetes in patients presenting to Blacktown/Mount Druitt emergency departments before and since the COVID-19 pandemic has begun, with a similar examination of diagnosed COVID-19 cases in GP clinics, as well as some interrogation of the reasons that this rate may have changed with a particular focus on a switch to VC. We hypothesize that the rates have varied substantially due to the pandemic, and that this has had an impact on the reasons for using VC.

## Methods

This analysis used an existing dataset of those who have had HbA_1c_ tests through the ED in Blacktown and Mount Druitt Hospitals in western Sydney. The detailed methodology for this testing has previously been published [8]. Briefly, all patients who attend the ED and have a blood test irrespective of reason for presentation undergo HbA_1c_ testing on the proviso that there is sufficient sample to undertake this measurement. Patients are not re-tested if they represent within 3 months of a previous test. This dataset represents 173,805 tests performed between 1^st^ June 2016 and 12^th^ November 2021. 75 HbA_1c_ tests were missing in this population, leaving 120,083 to be analysed in this study.

There were no specific inclusion criteria beyond being included in this dataset, and only patients with missing data on HbA_1c_ tests were excluded. This methodology allows for both those already diagnosed with diabetes and those newly diagnosed through this screening program to be included.

We also analysed a sample of all patients attending selected General Practice clinics in western Sydney. This represented the de-identified aggregate information of 188 individual clinics, and a total of 1.8 million patients. Within this dataset, we looked at the proportion of patients with diabetes attending GP clinics before and during the implementation of lockdown measures. This also included an examination of HbA_1c_ testing rates in GP clinics, as a measure of the care that was being offered during this time. Diabetes in this dataset is defined as a GP flag of diabetes, excluding gestational diabetes. The dataset included data from the start of collection for these 188 clinics, September 2019, until the most recent extract date at the end of October 2021. Prior to September 2019, a smaller number of GP clinics were included in this dataset, with about 130 as of the start of 2019.

Finally, we retrospectively reviewed routinely-collected hospital data from Blacktown/Mt Druitt hospitals, two hospitals in western Sydney with a total of 600 beds. We also reviewed the VC provision using routinely-collected data from the Western Sydney Diabetes (WSD) clinics, which service both of these hospitals.

WSD is an integrated care program spanning primary and secondary prevention, with an aim to both prevent people from getting diabetes, targeting high-risk individuals to improve their health, and preventing further complications for those who already have diabetes. WSD has a series of clinics including a joint case-conferencing service [12], complex diabetes clinics, post-discharge clinics, insulin stabilisation services, app-based healthcare provision, and various other VC services. These provide around 5,500 occasions of service to over 1,000 patients per year. Services were classified into either those provided by VC (including telehealth, audiovisual consultations, email, and mHealth) or those provided FTF.

All analysis was performed using Stata 15.1. In the hospital dataset, we computed the average weekly proportion of people with an HbA_1c_ meeting the American Diabetes Association criteria for a test consistent with diabetes [10] of 6.5% (48 mmol/mol), as well as the 95% confidence interval for this figure. We then compared this to the observed proportion of people presenting since the state of New South Wales (NSW) began its lockdown on the week beginning 16^th^ March. We also stratified this by age and sex to examine whether these factors were influencing the rate of diabetes in this population. We compared the average rate of diabetes in the population both using the 95% confidence intervals and performing a simple t-test comparing the rates before and during the pandemic to gauge statistical significance.

## Results

Demographics for the ED population are presented in Table 1. The mean age of the entire sample was 51 years, with the average age increasing with HbA_1c_. There was a slightly higher proportion of females to males in the sample, with 55% of the overall sample being female. Men made up a larger proportion of those with elevated HbA_1c_.

**Table 1:** Demographics by diabetes status

	No Diabetes	Pre-diabetes	Diabetes
All (n)	62,473 (52%)	36,751 (31%)	20,859 (17%)
Age^a^	42.3 (19.3)	59.1 (19.0)	64.2 (16.0)
HbA_1c_^a^			
HbA_1c_ (%)	5.3 (0.3)	6.0 (0.2)	8.0 (1.6)
HbA_1c_ (mmol/mol)	34	42	64
Sex^a^			
Female	36,321 (58.1%)	19,256 (52.4%)	10,151 (48.7%)
Male	26,152 (41.9%)	17,495 (47.6%)	10,708 (51.3%)
BMI (Kg/m^a^2)^a^	27.5 (7.2)	29.1 (8.0)	31.2 (8.8)
Aboriginality^a^			
Aboriginal or Torres Strait Islander	3559 (5.7%)	1275 (3.5%)	863 (4.1%)
Neither Aboriginal nor Torres Strait Islander	58,914 (94.3%)	35,476 (96.5%)	19,996 (95.9%)

^a^mean with standard deviation in brackets ^b^ proportion of total. Diabetes/pre-diabetes defined using ADA criteria of > 6.5% and 5.7–6.4% as above

In the time period since the week beginning March 16^th^, the number of tests performed in these EDs has declined, from an average just below 642 per week to 556 per week. This appears to correspond to a decline in the rate of ED attendances, with roughly half the expected attendances per week in late March and April of 2020 compared to 2019. There have also been differences in the people attending ED, with fewer people over the age of 65, and a decline in the proportion who are female. This is shown in Table 2.

**Table 2:** Comparison of before/after COVID values for testing for the entire time period

	Pre-COVID	During COVID
Tests per week	642	556
Median age	50	47
% male	45.0	46.4
% over 65 years old	29.5	26.9
% under 40 years old	37.0	40.9

**Fig. 1:**
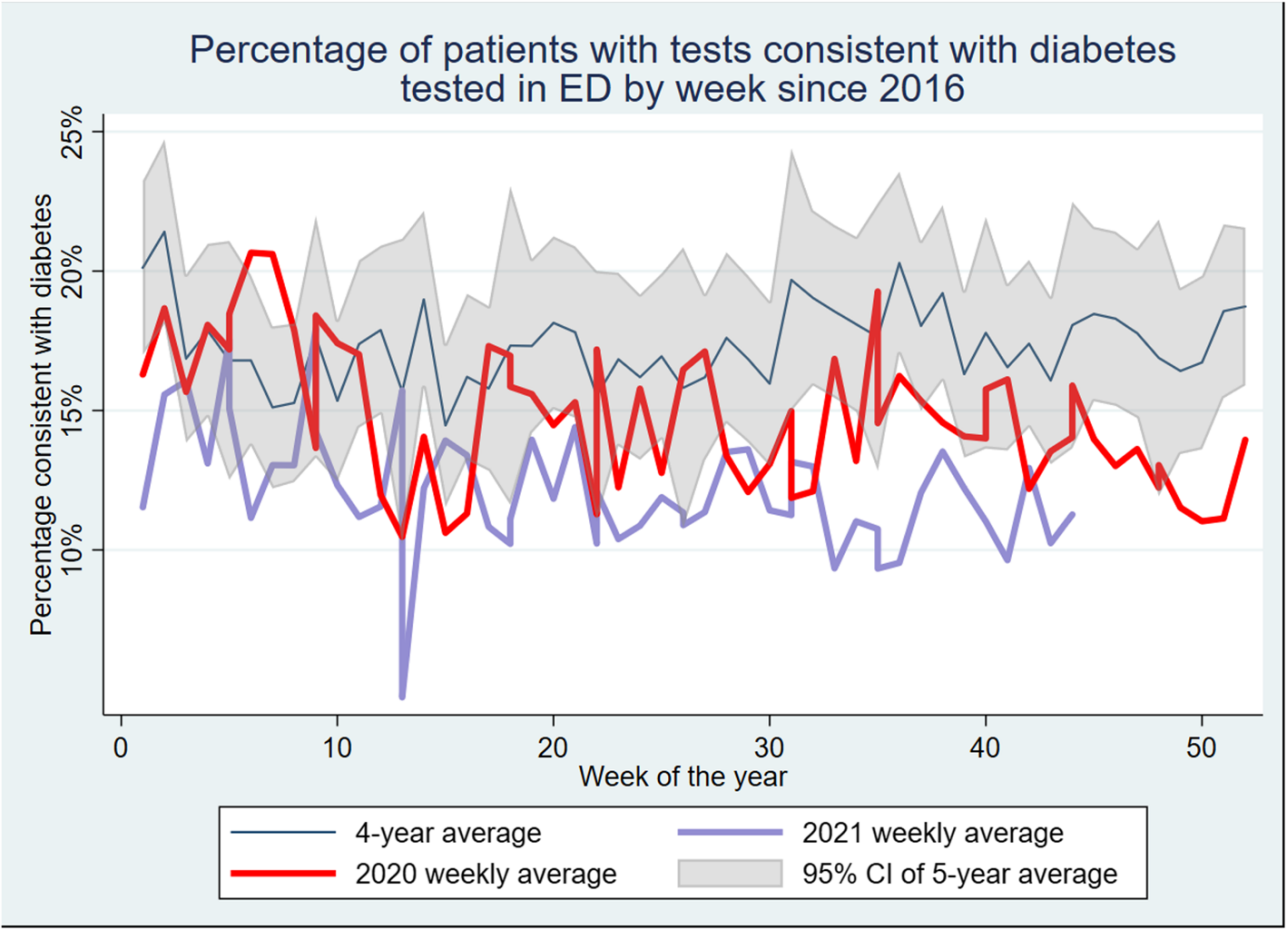
Percentage of patients with tests consistent with diabetes tested in ED by week since 2016. The 4-year average is for the years preceding the pandemic

The primary findings are presented in Fig. 1. During the time period from when NSW began legally enforcing isolation measures due to the epidemic of COVID-19, there was a significant decrease in the rate of people attending ED with elevated HbA_1c_ consistent with diabetes. As can be seen in Fig. 1, in most weeks this was below the 95% confidence interval from previous years. The rate has decreased from a mean varying between a low of 15% and a high of 25% to an average of just over 11% consistent with diabetes during this pandemic period.

**Fig. 2:**
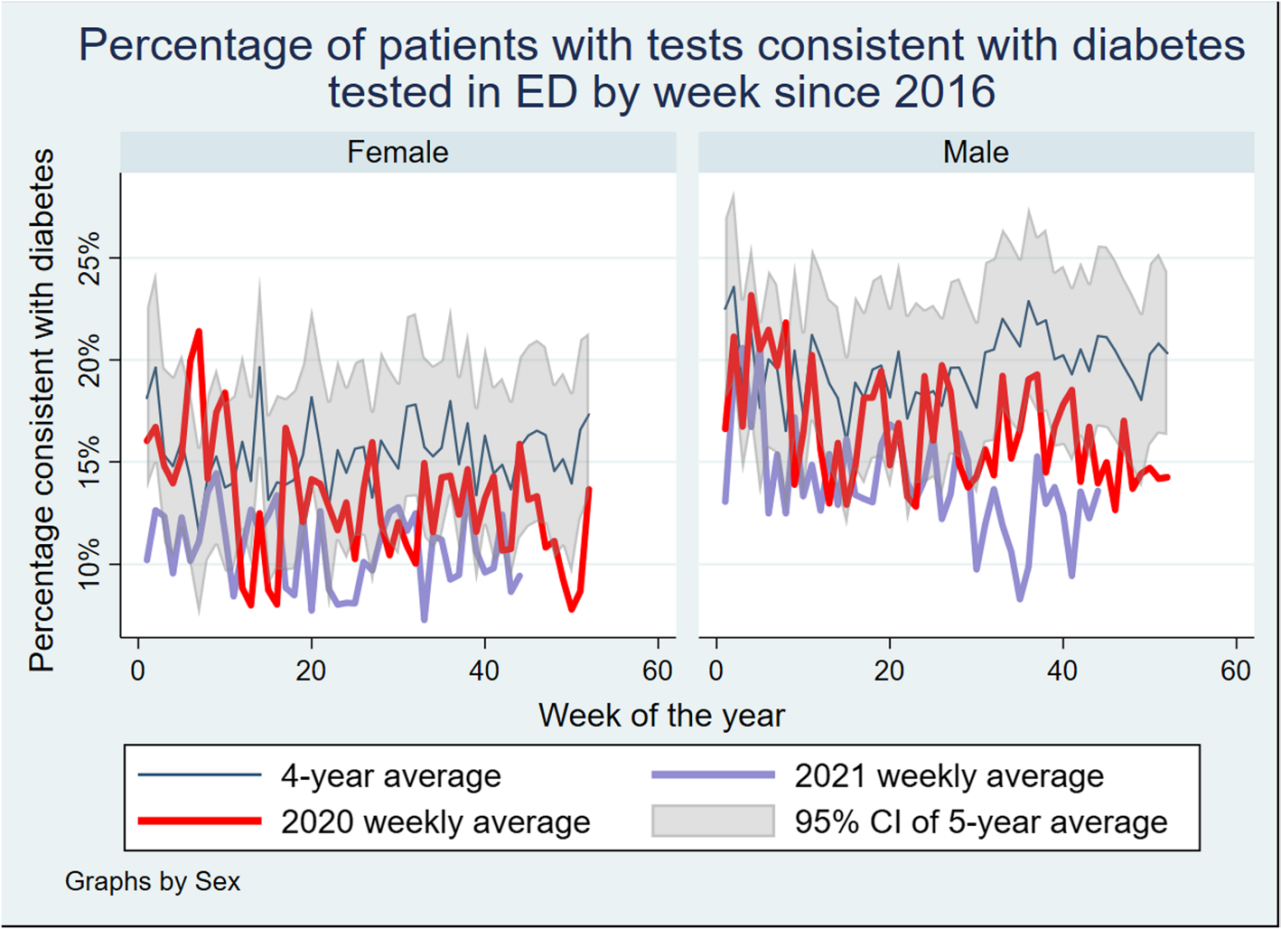
Percentage of patients with tests consistent with diabetes tested in ED by week since 2016 by sex

**Fig. 3:**
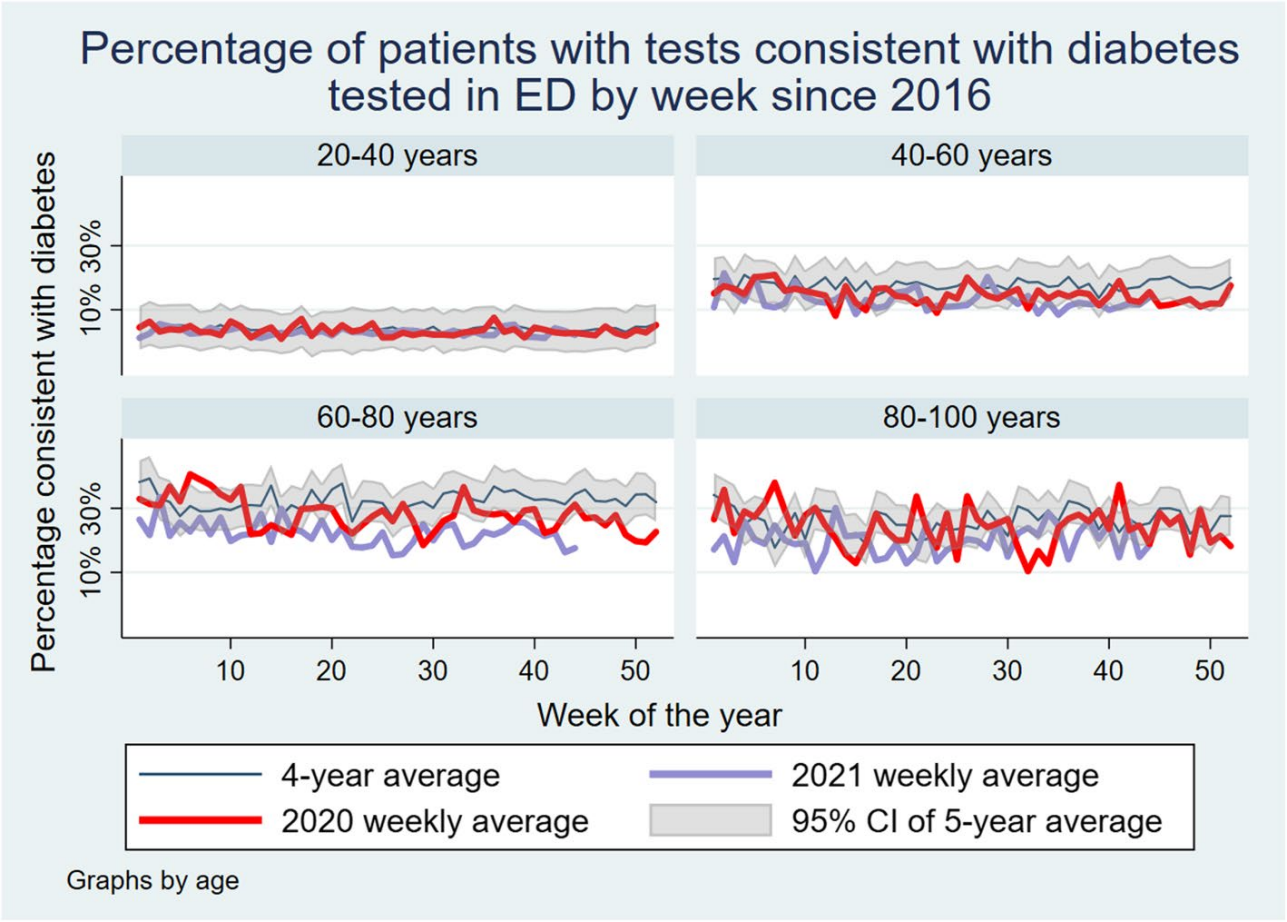
Percentage of patients with tests consistent with diabetes tested in ED by week since 2016 by age

Examining this rate by gender and age, there are some important findings. The reduction in the rate of tests consistent with diabetes appears to be largely driven by a decrease in the median age of people presenting. Once age-stratified, there appears to be some difference in diabetes percentages by age group. This is shown in Fig. 2. There is also indication of a significant interaction with gender, with females in this sample showing a downward trend in high Hba_1c_ results, however this trend was not apparent in the male population until the second wave in NSW. This is shown in Fig. 3.

Comparing the pre-COVID-19 rates of diabetes with the pandemic rate using t-test gives a *p*-value < 0.001, with the average rate of diabetes-consistent tests at 17.3% prior to COVID-19 and only 13.1% now.

**Fig. 4:**
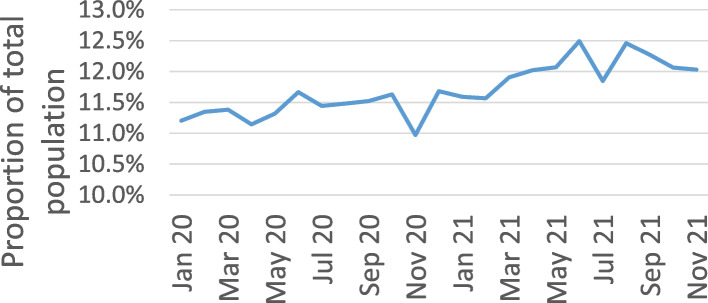
Proportion of adults attending GP clinics with diagnosed diabetes (*n* = 615 k per month)

These findings were not matched in the GP data. The mean rate of diabetes patient attendance in adults in the GP dataset prior to March in 2020 was 11.3%, which rose slightly to 11.8% during the pandemic period as seen in Fig. 4. This then continued to rise throughout the pandemic, with the rate stabilizing at 12% towards the end of 2021. However, the average number of adults active in the system fell slightly from 633,228 to 609,936, a drop of 3.7%.

Moreover, there did not appear to be a greater rate of diabetes in hospitalized adults, with similar trends both before and during the pandemic.

**Fig. 5:**
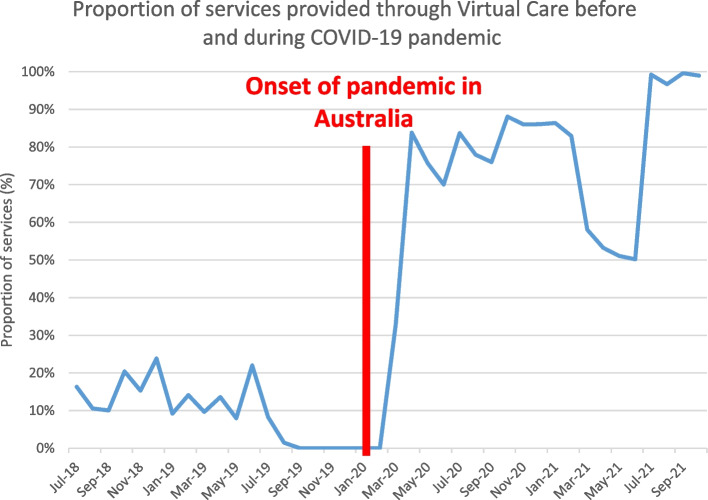
Proportion of services provided through Virtual Care before and during COVID-19 pandemic with onset of pandemic outlined in red

During the pandemic, the proportion of services offered through VC rose sharply from an average of 9.1% prior to March 2020 to 76.8% after that time (*p* < 0.0001), despite the average monthly number of consultations remaining steady during this time-period. This can be seen quite clearly in Fig. 5, which shows the stark divide between the proportion of services used before and during the pandemic. There was something of an initial lag during March/April 2020 as VC was being set up. Virtual Care had been temporarily halted at these clinics before March 2020 due to the setting up of a new service that was due to begin mid-2020 – the pandemic brought forward this new service substantially.

There was also a noticeable change in the proportion of services used when there were outbreaks compared to none. The first lockdown in NSW occurred at the point of the red line, with a second wave of restrictions coming through due to an outbreak in October 2020. These were relaxed in January 2020, at which point VC fell dramatically, and then reinstated during the 2021 lockdown in July 2021. Looking at the proportion of services provided through VC, where there were interventions in place to control outbreaks (April, May, June, November, December in 2020 and January, July, August, September, October in 2021) the average use of virtual care was 90.3% compared to 65.8% in non-outbreak months (*p* = 0.0014). All services continued to be provided during the pandemic period, although the denominator increased modestly as a new clinic was started in May 2020.

Hospital services appeared to be unaffected, with similar numbers pre and during pandemic for admissions due to hypoglycaemia, retinopathy, and diabetic chronic kidney disease (*p* > 0.05) in these hospitals.

**Fig. 6:**
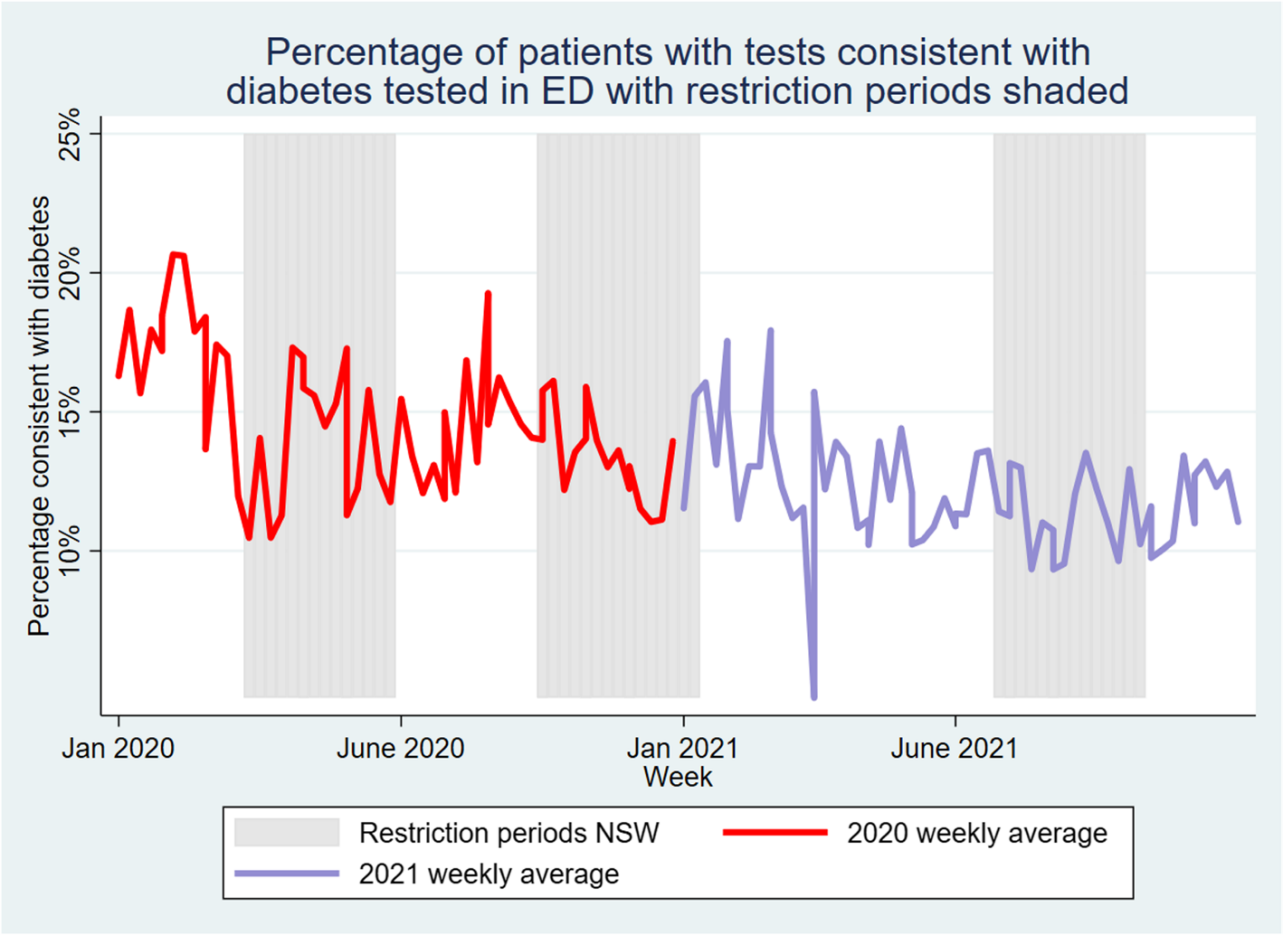
Proportion of those testing positive with lockdown periods shaded

Finally, there was an interesting trend whereby during periods where NSW experienced lockdowns (see Fig. 6), there were fewer patients who attended the ED with tests consistent with diabetes. Comparing the average rate of diabetes using a t-test between the times where the state was locked down and those it was not produces a *p*-value below 0.001.

## Discussion

In this study, we demonstrate that there is a significant trend in established datasets towards reduced proportion of people having tests consistent with diabetes in ED but not in GP. Indeed, in contrast to the effect seen in ED, there appear to be more people attending GP with diabetes in 2021 than the prior rate in adults during 2019/20. While this may be related to increased testing, it is also likely to be some evidence of replacement of services as people have avoided hospitals and instead attended GP clinics for their chronic disease during COVID-19.

Moreover, at the onset of the COVID-19 pandemic, there was a steep increase in use of VC services in two hospitals in western Sydney. From a very low baseline of less than 10% of services provided through VC, clinics pivoted to provide many or even most appointments through VC modalities. This has allowed these outpatient chronic care services to continue despite ongoing restrictions during the pandemic, and has no noticeable acutely negative impact on patient care.

This is similar to trends seen elsewhere during the pandemic. Numerous studies have demonstrated reduced use of hospital and GP services during lockdowns and other high-transmission periods internationally [13, 14]. Moreover, there has been a hypothesized impact on diabetes-specific services during COVID-19 from patients wary of infection or unable to attend services due to lockdowns [15, 16]. We have now proven that these worries may be well-founded, as people with diabetes have substantially reduced their interaction with hospital services during the pandemic, especially during high-transmission periods. Similarly large studies elsewhere in the world have found varying levels of concern over this important issue in the past [15].

These findings have important implications to current and future practice. While it may be difficult to manage diabetes during a pandemic, the fact that people with diabetes were on average a lower proportion of the population attending hospital and community services well before cases peaked in various waves has some potential negative connotations. While NSW began locking down on the 16^th^ of March, there were at this point few deaths in the state. However, there was a marked decline in both the number and proportion of people with HbA_1c_ consistent with diabetes attending ED, and fewer people attending their GP, perhaps indicating an undercurrent of fear in the general population of being infected with the virus, in terms of accessing health services. This was compounded by an additional ‘lockdown effect’, where people with diabetes were substantially less likely to attend ED during the state’s lockdowns. The concern here is that individuals with diabetes may become unwell independent of factors directly related to the pandemic; any delay in them presenting to hospital may result in a more severe and complicated illness.

However, there is also a positive reading of these data. If this represents a replacement of services, it may actually be a good outcome, by reducing the usage of high-cost tertiary services and pushing people towards more care in the community [12, 17]. This is also noticeable in the massively increased use of VC in hospital clinics that has continued even after restrictions were lifted.

This may also be seen in a positive light. During this time of increased activity within the health facilities preparing to combat COVID-19, a reduction in presentations of individuals with chronic disease to higher risk facilities is possibly ideal to reduce the risk of viral infection. The fact that GP attendance for the management of diabetes has not dropped, and appear to have increased slightly, may represent a shift towards telemedicine during this time, although the data is not yet in to demonstrate this.

However, this reluctance to attend ED and GP has led to the rapid development and maturation of services to support community based management with the availability of new funding through the Medicare Benefits Scheme (MBS) facilitating the process. In Blacktown Hospital, the majority of ambulatory care services, including clinics for complex type 2 diabetes have been converted from face-to-face encounters to telephone and telehealth services, which persisted in some form throughout the pandemic period. This includes the provision of video consultations for joint GP-specialist case conferencing and diabetes education, the establishment of pathways for flash glucose monitoring utilizing local pharmacies and a package of app-based interventions, to ensure that people with diabetes are still able to access care during this period.

Indeed, the potential replacement of ED with other service echoes international evidence demonstrating that patients have often switched from existing services to virtual care modalities to avoid in-person consultations during the pandemic [18]. This change in the use of healthcare services has the potential to improve diabetes care, insofar as it reduces reliance on high-cost emergency services and provides more sustainable chronic care for patients who have long-term chronic disease [19]. However, this approach may also have drawbacks – these primary care services do not always have sufficient resources to treat severe or complex cases [20], and the reduction in presentations to ED does not perfectly mirror the increases seen in GP. It is likely that some individuals have missed out on needed care even if some replacement took place, which may represent a burden in terms of untreated chronic disease as time goes on [4].

## Conclusions

Overall, we demonstrated that during a during the COVID-19 pandemic, the rate of presentations consistent with diabetes in a busy ED declined significantly from 17.4% to 13.1% per week. The rate of attendances to GP clinics for diabetes in the same area was not similarly impacted, with the proportion of patients diagnosed with diabetes actually increasing, however the total number of presentations was reduced. This was primarily driven by a smaller proportion of older patients presenting, with a younger median age in the group attending the ED than in previous periods and a significant age interaction with the trend, and may indicate a less acute patient population overall in the hospital outside of COVID-19 presentations. This complex interaction requires further exploration, and may have both costs and benefits for the healthcare system. A key future goal will be to identify whether people have replaced their missed ED care through GP services, or if this represents a worrying increase in diabetes service use in the healthcare system more broadly.

## Data Availability

The data that support the findings of this study are available from NSW Health but restrictions apply to the availability of these data, which were used under license for the current study, and so are not publicly available. Data may be available from GMK Gideon.meyerowitzkatz@health.nsw.gov.au upon reasonable request and with permission of NSW Health as well as relevant ethics bodies governing the use of this data for research and other purposes.
